# Beyond ritual bronzes: identifying multiple sources of highly radiogenic lead across Chinese history

**DOI:** 10.1038/s41598-018-30275-2

**Published:** 2018-08-06

**Authors:** Ruiliang Liu, Jessica Rawson, A. Mark Pollard

**Affiliations:** School of Archaeology, University of Oxford, 36 Beaumont Street, Oxford, OX1 2PG UK

## Abstract

One of the greatest enigmas in the study of Bronze Age China is the source of highly radiogenic lead discovered in the copper-based objects of the Shang period (ca. 1500–1046 BC). Although being relatively rare in nature, such lead contributed over half of the lead consumed across a vast area from the Yellow River to the Yangtze. Identifying its source and supply network would significantly contribute to our understanding of how China achieved the largest metal production across Eurasia. The past thirty years of research have seen various proposals for the origin of this lead, including south-western China, the middle Yangtze River valley, the Qinling and Zhongtiao mountains, and even Africa. This paper attempts to illustrate the tempero-spatial pattern of this highly radiogenic lead using the largest possible databank. Furthermore, by going beyond the bronze data and investigating lead isotopes in non-metal objects, we confirm that multiple sources of highly radiogenic lead must have been used across Chinese history. In turn, this implies the feasibility of a multi-source model for the lead in the Shang bronzes.

## Introduction

A major application of lead isotope geochemistry in archaeology has been in provenance studies, of which metal sources employed in the Mediterranean Bronze Age has undoubtedly attracted most attention^1,2^. Initially, archaeologists were encouraged because lead isotopes prove to be constant from ore to object. This offers a great advantage over the trace/minor elements, which can be dramatically altered during the process of mining, roasting, smelting and casting. Nevertheless, it was increasingly realized that sourcing metals by lead isotopes is not without difficulties, such as the overlap between different sources^3^, the variation of isotopes in a single source^4,5^, or the possibility of anthropogenic intervention (e.g., mixing and recycling)^6,7^.

**Figure 1:**
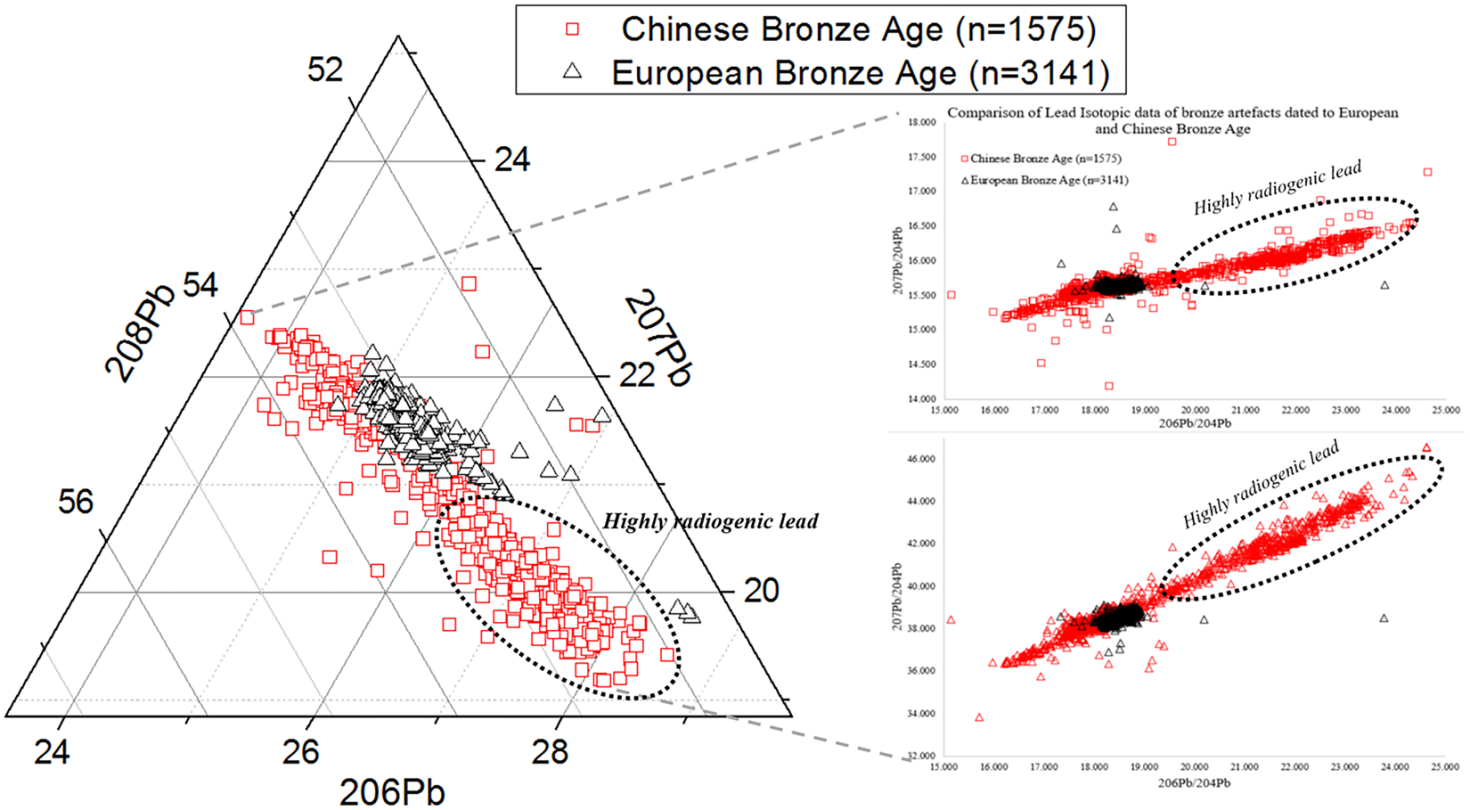
Comparing lead isotopic data in bronzes from Bronze Age Europe and China. The X-Y plot is the conventional way of illustrating lead isotope data, with ^206^Pb/^204^Pb against ^207^Pb/^204^Pb and ^208^Pb/^204^Pb, respectively; the ternary plot follows the method suggested in^31^ in order to present the full variation of the three radiogenic lead isotopes ^206^Pb, ^207^Pb and ^208^Pb. Original data in Oxford Archaeological Lead Isotope Database (http://oxalid.arch.ox.ac.uk) and the online supplementary material.

In recent decades, the discovery of highly radiogenic (anomalous) lead in ancient Chinese bronzes has again demonstrated the power of lead isotope studies in archaeometallurgy. A quick comparison of the isotopic ratio in bronzes between Europe and China shows a striking discrepancy in the nature of the lead (Fig. 1). The considerable variation in Chinese bronzes potentially offers a better opportunity to disentangle the interaction between Bronze Age societies and natural resources. Like many scientific methods in archaeology, however, the application of lead isotopes in the Chinese Bronze Age opens up more questions than answers. By broadening out the discussion of highly radiogenic lead in China from bronzes to other materials, this paper aims to suggest a possible way forward in solving the long-term question of where this lead came from.

## Lead isotopes in ores

Lead has four stable isotopes (^204^Pb, ^206^Pb, ^207^Pb, ^208^Pb), all of which were present at the formation of the Earth, but three of which (^206^Pb, ^207^Pb, ^208^Pb) are also produced by the radioactive decay of uranium and thorium (respectively ^238^U, ^235^U and ^232^Th). In the absence of unusually high concentrations of uranium and thorium, and in a closed geological environment, the isotope ratios of lead in a mineral deposit will evolve along predictable lines over geological time, and the geological age of such deposits can be calculated from a measurement of these isotope ratios^8,9^. Such deposits are termed *conformable* if they are describable by simple isotope evolutionary models, and they tend to have lead isotope ratios close to ^206^Pb/^204^Pb ≈ 18.5, ^207^Pb/^204^Pb ≈ 13.3, and ^208^Pb/^204^Pb ≈ 38.3. This is approximately the average lead isotopic composition in the Earth’s crust, and lead-containing minerals with such ratios are termed *common* or *ordinary* leads. The lead isotope ratios of minerals formed in the presence of excess uranium and/or thorium, however, evolve along different lines as a result of the production of additional amounts of ^206^Pb, ^207^Pb, and ^208^Pb, the exact enrichment depending on time and the amounts of uranium and thorium present. Such deposits are termed *non-conformable*, and their ages cannot be calculated from measurements of the lead isotope ratios using simple evolutionary models – they can give predicted (model) ages which are in the future and are generally referred to as *anomalous* (Joplin-type or J-type) leads. The other type of anomalous lead, Bleiberg type or B-type, formed in the early stages of the Earth’s evolution but has been remobilised, transported and re-emplaced at a later date and mixed with younger normal and/or radiogenic leads from sources with greater depletion in U/Pb than in Th/Pb (usually in the lower crust). The calculated model age of a B-type deposit is typically older than the true age of the secondary emplacement, in contrast to the J-type lead giving a younger age than the true age^10,11^. It is worth pointing out that, in addition to the original lead (^206^Pb = 18.8, ^207^Pb = 20.6 and ^208^Pb = 58.6 in atom percent, respectively^12^), *all* terrestrial lead deposits contain varying proportions of radiogenic lead. Therefore, the use of the term ‘radiogenic’ in many publications to describe anomalous lead is inappropriate. The term ‘highly radiogenic’ is more acceptable (e.g., ^206^Pb/^204^Pb >19.5), and has been used in some of the archaeological literature (and is used here). But ‘anomalous (Joplin-type) lead’ would be better since the ultimate characteristic of this type of lead is that it produces negative (future) ages in the Holmes-Houtermans or Stacy-Kramers models^13–15^.

In practice, using lead isotopes to characterize metal deposits is not a straightforward task. Very few lead deposits are truly conformable, but, conversely, deposits with highly radiogenic lead isotope ratios are relatively rare. Moreover, deposits with highly radiogenic leads can be very heterogeneous. Famously, Cannon *et al*. reported isotopic measurements on a single 10 cm cubic crystal of galena (PbS) from the Tri-State District in the Mississippi Valley (USA) which showed half the total variation of the entire deposit (^206^Pb/^204^Pb = 21.91–22.92; ^207^Pb/^204^Pb = 15.99–16.11; ^208^Pb/^204^Pb = 41.17–42.02^16^), with the least radiogenic values at the centre of the crystal. This they interpreted as mixing of lead with an increasingly radiogenic signature in the ore-forming fluid during crystallization. There may therefore be systematic zoning within the deposit, and anomalous lead ores may be very localized, occurring within deposits showing otherwise ‘common’ values of lead.

### Lead isotopes in ancient Chinese bronzes

**Figure 2:**
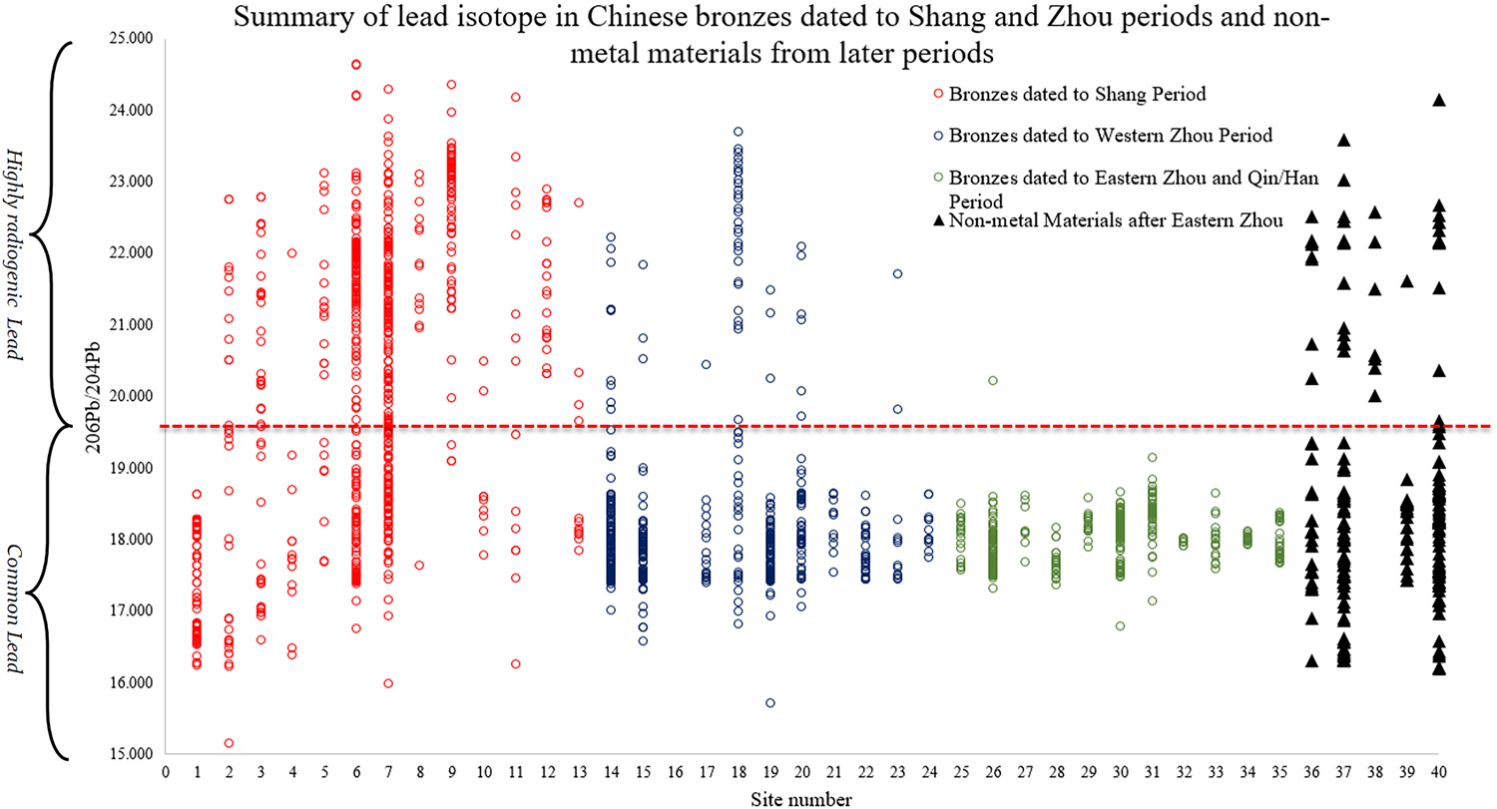
Rise and fall of highly radiogenic lead in Chinese history (n = 1907; 1. Erlitou 2. Zhengzhou 3. Panlongcheng 4. Yuanqu 5. Hanzhong (Early Shang) 6. Anyang 7. Northern Shaanxi (Late Shang) 8. Hanzhong (Late Shang) 9. Sanxingdui. 10. Haimenkou 11. Yueyang bronzes 12. Xin’gan 13. Northern Steppe Late Shang 14. Zhouyuan (Sackler Western Zhou) 15. Zeng State 16. Yu State 17. Guo State 18. Jinsha 19. Jin State 20. Tanheli-Gaoshaji 21. Northern Shaanxi Western Zhou 22. Yan State 23. Jiang State 24. Shuangyantai 25. Northern Shaanxi (Eastern Zhou) 26. Sackler (Eastern Zhou) 27. Yue bronzes 28. Changzhi Fenshuiling 29. Chongqing bronzes 30. Upper Xiajiadian 31. Yunnan bronzes 32. Xiaxiangpu 33. Liu’an 34. Qishihuang mausolin 35. Han mirrors 36. Non-metal materials Zhou dynasty 37. Non-metal materials Qin-Han 38. Non-metal materials Western Wei 39. Non-metal materials Tang to Qing 40. Chronology unknown).

**Figure 3:**
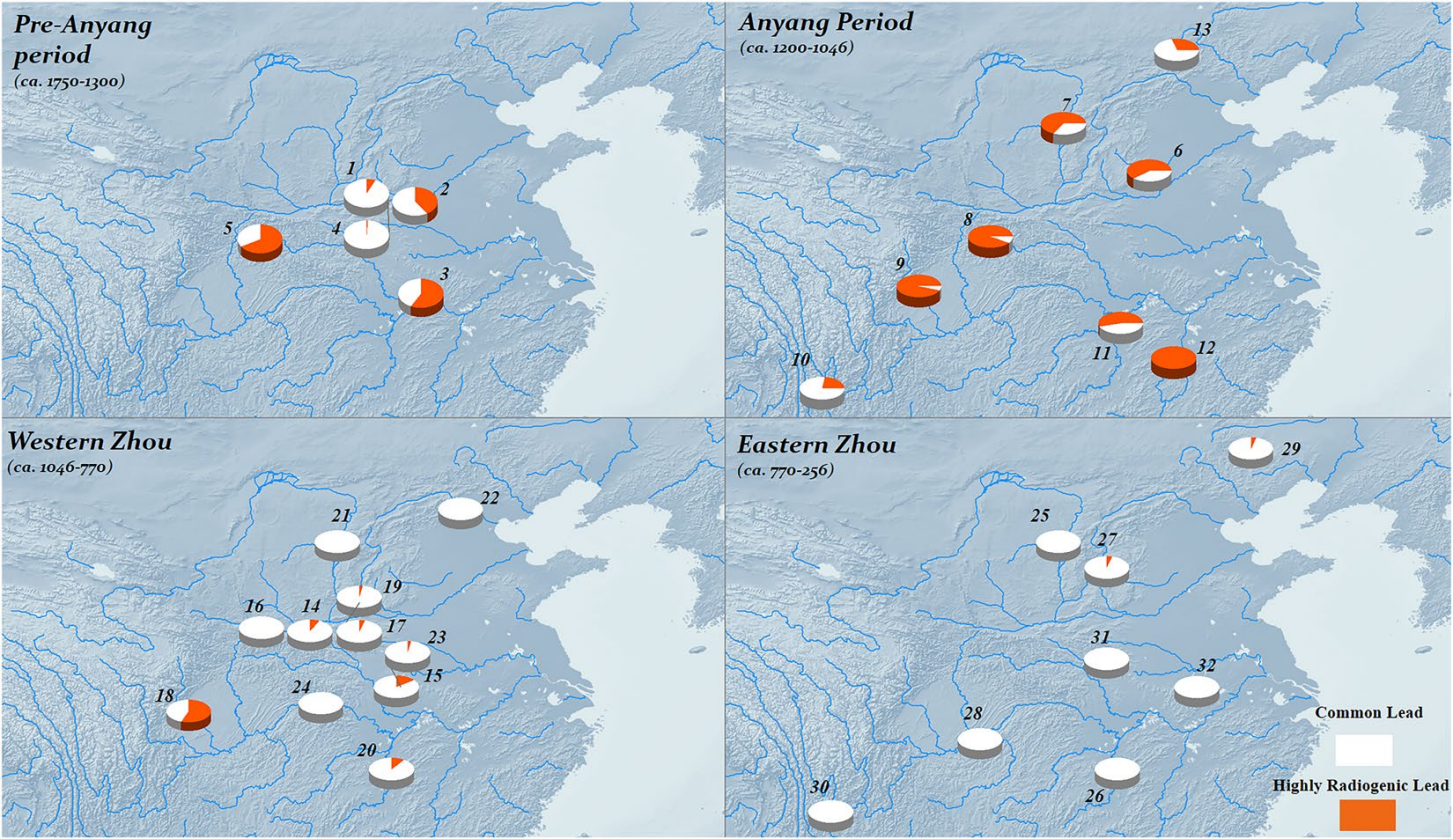
Distribution of highly radiogenic lead in bronzes of Shang and Zhou China (1. Erlitou 2. Zhengzhou 3. Panlongcheng 4. Yuanqu 5. Hanzhong (Early Shang) 6. Anyang 7. Northern Shaanxi (Late Shang) 8. Hanzhong (Late Shang) 9. Sanxingdui 10. Haimenkou 11. Yueyang bronzes 12. Xin’gan 13. Northern Steppe (Late Shang) 14. Zhouyuan 15. Zeng State 16. Yu State 17. Guo State 18. Jinsha 19. Jin State 20. Tanheli-Gaoshaji 21. Northern Shaanxi Western Zhou 22. Yan State 23. Jiang State 24. Shuangyantai 25. Northern Shaanxi (Eastern Zhou) 26. Yue bronzes 27. Changzhi Fenshuiling 28. Chongqing bronzes 29. Upper Xiajiadian 30. Yunnan bronzes 31. Xiaxiangpu 32. Liu’an; the satellite imagery generated by ArcGIS 10.4.1, https://desktop.arcgis.com/en/quick-start-guides/10.4/arcgis-desktop-quick-start-guide.htm).

More than thirty years have passed since the first report of the finding of highly radiogenic lead in Shang Dynasty Chinese bronzes^17^. Since then, the use of highly radiogenic lead in Bronze Age China has been frequently mentioned and discussed in many important publications^18–20^. It is only recently that detailed attention has been paid to this long-standing issue of Chinese archaeology in the English language literature^21,22^. The most recent and high profile debate on Chinese highly radiogenic lead was triggered by Sun *et al*., who argued that the metal contained in the Chinese ritual bronze vessels unearthed at Anyang originated in Africa^23^. Although extremely eye-catching, this paper provided little evidence which can be considered archaeologically valid^24^. A comprehensive overview of this subject has been offered by the FLAME team^21^, which shows the widespread use of highly radiogenic lead in Chinese ritual bronzes from the Erligang (Zhengzhou) period (ca. 1500–1300 BC) until the Yinxu II Period of Anyang (ca. 1200–1046 BC). This remarkable phenomenon, as shown in Figs 2 and 3, shows that for a period of about 200 years, much of the lead used in the casting of these bronzes was highly radiogenic in origin. Even more remarkably, isotopic measurements of bronzes from outside the Central Plains of China – that is, from Sanxingdui in Sichuan, from Panlongcheng and Xin’gan on the Middle Yangtze River – have shown the virtually simultaneous use of highly radiogenic lead in their cast bronzes. Equally intriguing and mysterious, it appears to have disappeared in the subsequent Zhou dynasty (Fig. 3).

**Figure 4:**
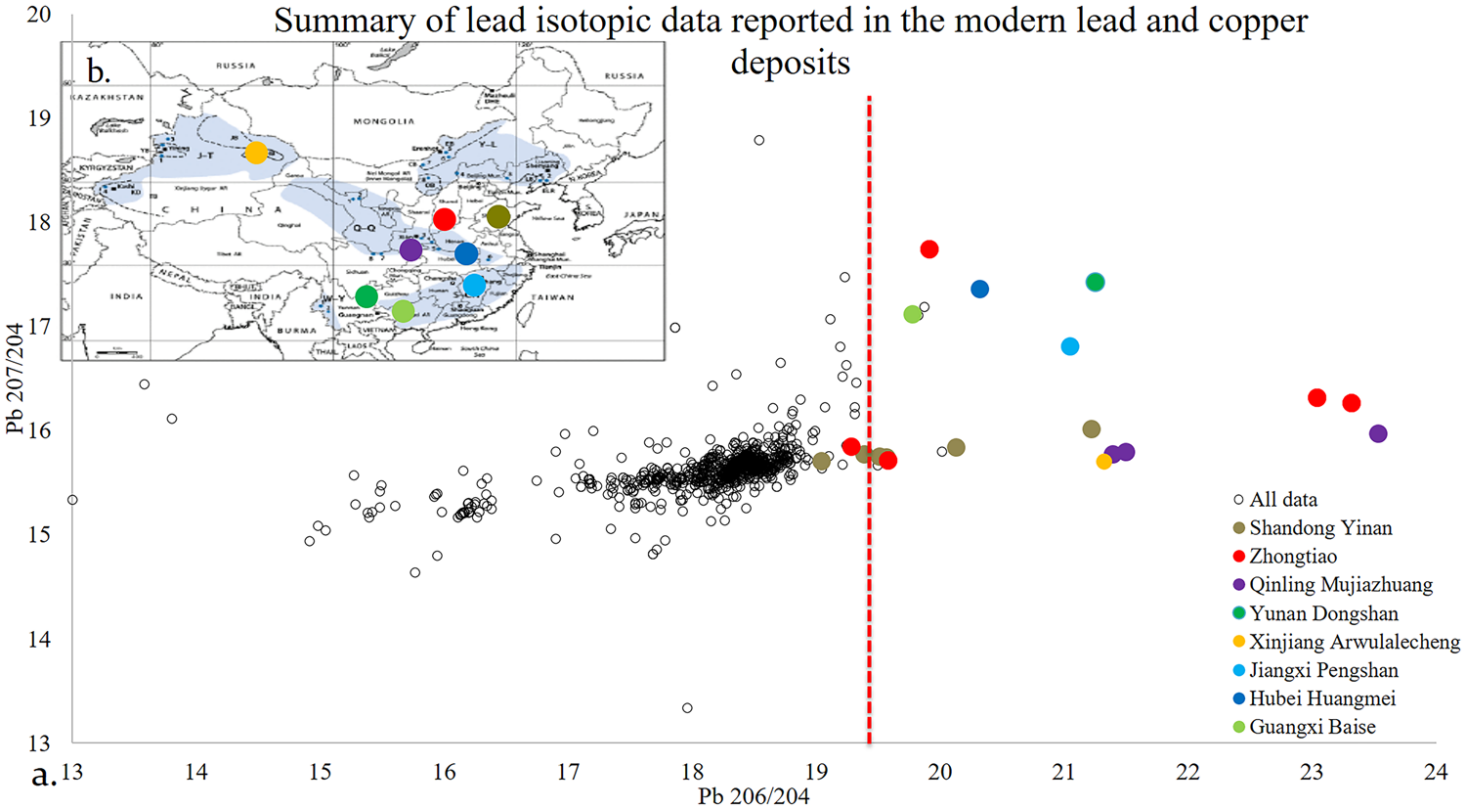
(**a**) Summary of Lead Isotopic Data of modern copper/lead deposits in China^24^, (**b**) copper/lead deposits containing highly radiogenic lead with the base map of uranium distribution in China from^32^.

Consideration of the source of highly radiogenic lead in bronzes has occupied Chinese archaeologists and archaeometallurgists for around thirty years. As summarized by FLAME^21^, modern geological data reveals a number of regions of China in theory capable of supplying highly radiogenic lead, including the middle Yangtze River, the Qingling and Zhongtiao mountains, and, most famously (since it was first suggested), the south-western part of China, around Northern Yunnan (Fig. 4). Apart from various pitfalls of comparing data of the modern ores to archaeological objects^25^, it is also crucial to note that *source* here is used as an analytical term. Although it conveys different meanings on varying scales (from vast metallurgical province, or a large metalliferous belt, to a specific ore body, or even to a small specific mine shaft)^26^, in this paper, it is primarily used to refer to broad regions where major copper/lead mines occur ubiquitously, such as the middle and lower Yangtze River Valley^27^. It can be anticipated, however, that the ongoing mining archaeology studies in China may provide a picture with better resolution in the near future.

Whilst the exact source of such lead has so far eluded definitive identification, perhaps more interesting is the dilemma which the widespread observation of this highly radiogenic lead poses for archaeologists. We must assume that Bronze Age metallurgical workers in China (as elsewhere) had no means of distinguishing lead with anomalous isotopic values, beyond the unwitting selection of such metal by the preferential use of raw materials from a particular source. In this case, the observation that all of the major sites approximately contemporary with Shang, stretching from the Yangtze River to the Central Plains, used highly radiogenic lead for a maximum period of three hundred years is in itself remarkable. This *widespread* and *synchronous* pattern has at least two possible explanations:

(i) Highly radiogenic lead was exploited from a single source, accessible by a variety of local powers, of which the Shang was probably the most important, and it was subsequently distributed across a supply network controlled by the Shang, or:
(ii) There were multiple sources of highly radiogenic lead exploited between 1400 and 1200 BCE, accessibly by many groups of people across a vast area, of which the supply mechanism remains unknown.

Neither of these explanations appears particularly convincing. Variations are possible between these two extremes, such as the Shang empire exploiting more than one source of highly radiogenic lead, but, nevertheless, the basic conundrum remains – *how and why did bronze casters over a large region synchronously start and stop using radiogenic lead, when they had no means of identifying such lead?*

We argue that it is unlikely that the solution to this dilemma, let alone the identification of the source(s) of such lead, will be revealed simply by analysing more bronzes from the Chinese Bronze Age, beneficial though that would be for other reasons. Further elucidation may come from looking at the isotopic ratios of lead in bronzes from later periods, and, more critically, from lead in other materials, which is what this paper sets out to do. We think that the debate centred on highly radiogenic lead should consider not only where this lead came from, but also how it became so accessible to a range of regional powers during the same period. In other words, it is not necessarily a dilemma but more an opportunity to further our understanding. The occurrence of highly radiogenic lead is clearly a potential aid to provenance, but, equally importantly, provides a sensitive proxy for metal movement, human interactions and social organization.

### Lead isotopes in Chinese materials other than Bronze

Through a large survey of the existing published literature in various languages, we have expanded our Chinese lead isotope database to include non-metal materials (n = 259) which contain varying amounts of lead, including glass, leaded glaze and pigments (Data listed in Online supplementary material). There are three questions to consider:

1. Do these non-metallic materials contain highly radiogenic lead?
2. If so, what temporal/spatial patterns can be illustrated by these data?
3. Is it possible to further infer the provenance of such lead from these data?

Surprisingly, the total number of the lead isotopic data for all non-metal materials (n = 259), relative to that of bronzes (n = 1648), is rather small. More disappointingly, the majority of these data are from unprovenanced objects, making it extremely difficult to address questions 2 and 3 above. The lack of secure archaeological context severely limits the potential information that can be drawn from these data.

However, at a broad level, there are still some important observations to be made. Undoubtedly, the use of anomalous lead is not a unique feature of bronzes. The right-hand side of Fig. 2 shows that, for a variety of periods and dynasties, almost every kind of non-metal material contains anomalous lead. It is therefore surprising that there is very little discussion of its presence in Chinese glass beads/faience, leaded glazes, or pigments in the primary literature. Nevertheless, this finding, when connected to the data on ritual bronzes in the Shang period, is extraordinary (Fig. 2).

Whilst highly radiogenic lead decreases markedly in bronzes after phase III at Anyang^21^, it is clear that it continues to be used in vitreous materials and pigments through the Warring States, Han, Three Kingdoms and Western Wei, only seeming to disappear in the Tang and later material (but note that this observation is based on a very limited number of samples). Three subsets of data quickly attract our attention, not only because of the highly radiogenic signatures they show but also because the locations of the material are recorded in the original literature.

### Pigments from Tianlongshan Cave

**Figure 5:**
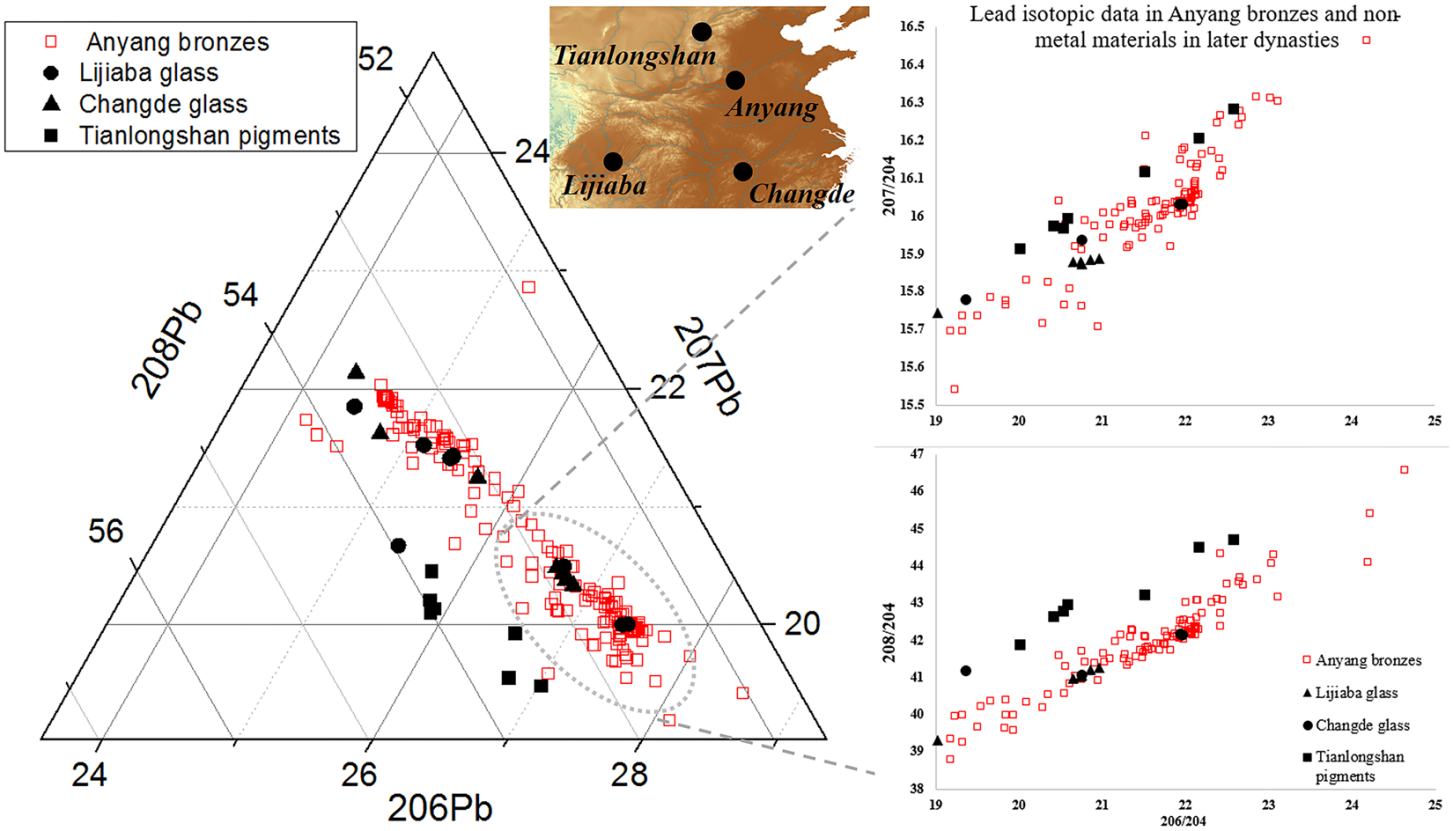
Discoveries of highly radiogenic lead in non-materials of post Chinese Bronze Age (the satellite imagery generated by ArcGIS 10.4.1, https://desktop.arcgis.com/en/quick-start-guides/10.4/arcgis-desktop-quick-start-guide.htm).

Brill *et al*. have reported lead isotopic data in seven black pigments (plattnerite, PbO_2_) from different locations in the Buddhist wall paintings of the Tianlongshan cave in present-day Shanxi province (Fig. 5)^28^. The paintings are stylistically dated to the Western Wei dynasty (AD 537–557), with no obvious signs of being repaired or repainted. All seven analyses indicate the use of highly radiogenic lead (Fig. 5). This was also noted by Brill and colleagues but articulated there in different terms^28^. Considering the large scatter between individual results from these seven samples, Brill and colleagues suggested that more than one source of lead could be involved, and that mixing had probably occurred. They also speculated that unnoticed repainting may also have taken place. They further commented that “the Tien Lung Shan (Tianlongshan) leads are a new type of lead to us. Except for the Chinese glasses, the authors know of no parallels”. In fact, this observation is supported by the comparison shown in Fig. 5, where these data appear rather different from the highly radiogenic leads of the Shang bronzes, particularly in the higher values of ^208^Pb/^204^Pb. To date, this is the first clear evidence for a source of radiogenic different to that of Shang bronzes. However, no direct information has as yet been found enabling us to associate the pigments with any ore source.

### Glass from the Chu State

The other two provenanced subsets of data are glass materials. The isotopic analyses of eleven glass objects from the tombs of the Chu state (Warring States period, 475–221 BC) in Changde (Hunan province) have been published by Cui *et al*.^29^. In spite of the typological differences (Bi/disk-shaped wares and eye beads), all of these samples are lead-barium glass, which is a technology unique to China. It is interesting to see that three out of the eleven samples show a highly radiogenic signature, and that all eleven isotope ratios coincide with the data from the Shang bronzes (Fig. 5).

### Glass from the Lijiaba site

The data by Cui *et al*. from another set of eight glass-based objects (seven ear pendants and one eye bead dated to the Eastern Zhou period) unearthed from the site of Lijiaba in the Sichuan Basin are also considerably important. They are all lead-barium glass, and five of them contain highly radiogenic lead^30^. The Sichuan basin in the Bronze Age is well-known for the use of such lead in bronzes. Over half of the bronzes discovered at Sanxingdui have been found to contain highly radiogenic lead, and the slightly later Jinsha site is so far the latest Bronze Age site in China which shows a dominant proportion of such lead. Its occurrence in these glass objects therefore undoubtedly extends this picture into the later period.

## Discussion and Conclusions

Scholars have been debating since the last century the meaning of the presence of highly radiogenic lead in the Shang Bronzes, specifically whether a single source or many sources are represented, and where these sources might be. Whilst it is true to say that the addition of new data from later non-metallic materials does not yet emphatically resolve this issue, it does add further evidence which potentially shifts the balance of the debate. From the data shown above, the presence of highly radiogenic lead in China is *not* a fleeting phenomenon seen *only* in Shang dynasty bronzes. It continues in other materials until at least the Tang dynasty. The paucity of analyses of bronzes from the Warring States onwards does not yet allow us to know if it becomes a characteristic of vitreous materials only, or whether it does in fact continue in later bronzes. The fact that these later vitreous materials, where we have provenance information, originate from all over China strongly suggests that multiple sources of highly radiogenic lead were available to, and indeed used by, these artisans. That this statement probably also applies to metal craftsmen in the Bronze Age is supported by the observation that it is not only the bronzes of the Central Plains which show highly radiogenic lead, but that contemporary metal objects from elsewhere also show such a signal.

This argument mitigates against the idea that highly radiogenic lead in Bronze Age China was procured from one source region, presumably under the control of the Shang, and distributed to other metal consumers. It does, however, beg the question of how metalworkers across China *simultaneously* started to use highly radiogenic lead, and, more critically, stopped using it at almost the same time. If we believe that, as is the case in Europe, sources of highly radiogenic lead are rare compared to those of common leads, and that ancient craftspeople had no ability to differentiate the two sorts of lead, then a simple combined probability argument suggests that the likelihood of more than one highly radiogenic source being independently discovered and extracted at the same time appears virtually impossible. As has been shown by a synthesis of the modern geological data (Fig. 4), however, highly radiogenic lead is sporadically available across all of the metalliferous mining regions in China. Multiple sources of highly radiogenic lead could certainly have been available from many of the mining regions exploited during the Bronze Age, although we must await further field data before we can be certain where and when such ores were exploited.

Importantly, we need to examine more carefully the assertion that bronze workers all over Bronze Age China *simultaneously* adopted and then rejected highly radiogenic lead. The only region for which sufficient data exists over an extended time period is, of course, the Central Plains. This shows the absence of anomalous lead during the Erlitou period (ca. 1750–1500 BC), the adoption of highly radiogenic lead in the Erligang (Zhengzhou) phase of the Early Shang (ca. 1500–1300 BC), and the decline towards the end of the Yinxu III phase at Anyang^21^. For all other areas, we simply have a temporal snapshot which shows the use of highly radiogenic lead during a period broadly contemporary to that of the Shang, based on the typologies of bronze and pottery. Very little data exist before or after this period (exceptions include Jinsha in early Western Zhou). So in fact all we can be really sure of is that there was a short period in the Central Plains during which highly radiogenic lead was used, and that it was also heavily used in other areas at approximately the same time. This is consistent with the observation that multiple sources of anomalous lead were available across Chinese history, and does not – until further data are available – require an explanation for the assumed synchronicity.

In summary, the isotopic data of non-metal objects in later periods has greatly expanded the occurrence of highly radiogenic lead in archaeological materials, both temporally and geographically. This points to the exploitation of multiple sources of highly radiogenic lead across Chinese history to produce various kinds of objects, not only the ritual bronzes of the Shang, but also pigments, leaded glaze and glass in later periods. Notably, the lead isotope data of the non-metal objects, although admittedly as yet few in number, show a surprising consistency with the picture from the earlier bronzes. Although it does not solve the long-standing question of the origin of such lead in Shang bronzes, it does put the question into a much broader perspective. A combination of further geological and archaeological fieldwork is required to identify these sources exploited in antiquity. Moreover, a programme of chemical and isotopic analysis of more well-provenanced material of all types is required, to include later bronzes.

## Electronic supplementary material

Supplementary Information
